# Flemish critical care nurses’ experiences regarding the influence of work-related demands on their health: a descriptive interpretive qualitative study

**DOI:** 10.1186/s12912-024-02032-6

**Published:** 2024-06-06

**Authors:** Lukas Billiau, Larissa Bolliger, Els clays, Kristof Eeckloo, Margo Ketels

**Affiliations:** 1 Strategic Policy Cell, Ghent University Hospital, Corneel Heymanslaan 10, Ghent, 9000 Belgium; 2 Faculty of Medicine and Health Sciences, Department of Public Health and Primary Care, Ghent University, Corneel Heymanslaan 10, Ghent, 9000 Belgium

**Keywords:** Critical care, Nursing, Psychosocial, Qualitative research, Sustainable employment, Work-related demands

## Abstract

**Background:**

Critical care nurses (CCNs) around the globe face other health challenges compared to their peers in general hospital nursing. Moreover, the nursing workforce grapples with persistent staffing shortages. In light of these circumstances, developing a sustainable work environment is imperative to retain the current nursing workforce. Consequently, this study aimed to gain insight into the recalled experiences of CCNs in dealing with the physical and psychosocial influences of work-related demands on their health while examining the environments in which they operate. The second aim was to explore the complex social and psychological processes through which CCNs navigate these work-related demands across various CCN wards.

**Methods:**

A qualitative study following Thorne’s interpretive descriptive approach was conducted. From October 2022 to April 2023, six focus groups were organised. Data from a diverse sample of 27 Flemish CCNs engaged in physically demanding roles from three CCN wards were collected. The Qualitative Analysis Guide of Leuven was applied to support the constant comparison process.

**Results:**

Participants reported being exposed to occupational physical activity, emotional, quantitative, and cognitive work-related demands, adverse patient behaviour, and poor working time quality. Exposure to these work-related demands was perceived as harmful, potentially resulting in physical, mental, and psychosomatic strain, as well as an increased turnover intention. In response to these demands, participants employed various strategies for mitigation, including seeking social support, exerting control over their work, utilising appropriate equipment, recognising rewards, and engaging in leisure-time physical activity.

**Conclusions:**

CCNs’ health is challenged by work-related demands that are not entirely covered by the traditional quantitative frameworks used in research on psychologically healthy work. Therefore, future studies should focus on improving such frameworks by exploring the role of psychosocial and organisational factors in more detail. This study has important implications for workplace health promotion with a view on preventing work absenteeism and drop-out in the long run, as it offers strong arguments to promote sufficient risk management strategies, schedule flexibility, uninterrupted off-job recovery time, and positive management, which can prolong the well-being and sustainable careers of the CCN workforce.

## Background

Globally, the nursing profession is a strenuous occupation with high levels of work-related demands, leading to adverse health outcomes for nurses [1], reduced marital and life satisfaction [2], absenteeism, and high costs for society [3]. In addition, the nursing workforce has to address staffing shortages due to the reduced number of individuals entering the nursing profession [4], the ageing working population [5], and the increased number of nurses in premature retirement [6, 7].

Especially critical care nurses (CCNs), who specialise in managing life-threatening diseases across all age groups, work in an exceptionally demanding environment [8]. Increasing evidence suggests that CCNs’ health is mainly challenged by five work-related demands, namely, occupational physical activity (OPA) [1], shiftwork [9], and quantitative [10], cognitive [11], and emotional work-related demands [12]. Among CCNs, OPA involves various physically demanding tasks, such as forward bending and isometric neck postures, heavy lifting, prolonged standing, and long-distance walking [1, 9]. With continued exposure to OPA, musculoskeletal disorders can arise in terms of pain-related complaints of the wrists, back, thigh, knees, and feet [1]. However, many studies have reported that engaging in regular leisure-time physical activity has a beneficial influence on health, while OPA may have no beneficial, or even adverse, influence on health [13]. These conflicting health influences are indicated as the “physical activity health paradox” [13] and might be explained by differences in duration, intensity, recovery opportunities, and physiological responses [14, 15].

In addition to OPA is shift work, which is the amount of time an individual works outside the typical nine AM to five PM schedule, known to impact CCNs’ health through circadian rhythm disruption, fatigue, and social isolation [16–18]. First, circadian rhythm disruption induces the proliferation of dysfunctional immune cells and is likely to cause cancer [19], coronary heart disease [20], diabetes mellitus [21], and gastrointestinal disorders [18, 22]. Second, fatigue may contribute to the development of cancer [16], coronary heart disease, diabetes mellitus, gastrointestinal disorders [23], and psychological stress [18, 24]. Finally, CCNs report experiencing social isolation because shift work makes it difficult for them to participate in leisure-time activities or family time, which can lead to depression [25, 26].

Furthermore, CCNs face quantitative work-related demands regarding high workload, time pressure, and workflow interruptions [10, 27]. These demands impair CCNs’ mental focus and increase the likelihood of developing prolonged fatigue and stress [10]. In addition, CCNs need to deal with high levels of cognitive work-related demands, which can be defined as: *“burdens placed on the brain processes involved in information processing”* [28, p.1574]. These cognitive work-related demands above the acceptable threshold contribute to attention narrowing, psychological stress, and burnout [29–31]. Moreover, CCNs are exposed to emotional work-related demands that require them to exert effort to deal with the desired emotional responses [28]. These demands involve workplace violence and end-of-life care issues and can cause anxiety, fatigue, and depression [12, 32].

Given the number of studies having postulated the adverse health effects of work-related demands, there is an increasing need for developing mitigating strategies to guarantee extended healthy working lives [33]. From a theoretical perspective, the Job Demand-Control-Support model [34] hypothesises job control and workplace social support as psychosocial moderators to mitigate the strenuous impact of work-related demands on health [35]. In particular, job control refers to: *“a working individual’s potential control over his task and his conduct during the working day”* [36, pp. 289–290]. It has been argued that job control can reduce the physiological impact of work-related demands on employees’ health by allowing them to take a break if necessary [35]. Likewise, workplace social support can be considered as interpersonal relationships at work to cope with stressful situations by putting them into another perspective, thereby leading to less psychological stress [37]. Additionally, the Effort-Reward Imbalance model [38] considers the prevention of adverse health outcomes by providing sufficient rewards in line with the performed efforts at work [39].

Numerous correlational studies are available which research the impact of work-related demands on nurses’ health [40–42]. To our knowledge, no qualitative studies have comprehensively investigated how exposure to multiple work-related demands influences CCNs’ health, or the complex social and psychological processes through which CCNs navigate these work-related demands across various CCN wards. However, it is essential to identify new factors in the research of CCNs’ work-related health and to create a policy that prevents health complaints and their associated costs.

## Methods

### The aims and design of the study

This qualitative study was based on Thorne’s interpretive descriptive approach [43] and was part of the Flemish Employees’ Physical Activity study [44]. Thorne’s interpretive descriptive approach embraces the concept that reality is shaped by social constructs, acknowledging the existence of diverse constructed realities [43]. Thus, this approach was appropriate to gain insight into the recalled experiences of CCNs in dealing with the physical and psychological influence of work-related demands on their health, while also examining the environments in which they operate [43]. In addition, this approach was well suited to explore the complex social and psychological processes through which CCNs navigate these work-related hazards across various CCN wards [43].

### Setting and participants

This study was conducted in a local hospital in Flanders (Belgium) with a capacity of 1046 beds. First, 18 CCNs were recruited between October 2022 and January 2023 by means of convenience sampling to ensure a wide range of experiences by posting recruitment flyers in the CCNs’ lockers and placing posters in the CCN wards. Moreover, an invitation mail with informed consent was sent to the head nurses, who then delivered this mail to their CCNs. However, the CCNs could also participate by directly expressing their willingness to engage by email to the research team. Eligibility criteria required CCNs to be employed for more than 50% in the emergency department (ED), intensive care unit (ICU), stroke unit, or the critical care mobile nursing team and to be Dutch speaking. Nurses of the critical care mobile nursing team were employed simultaneously in the ED, ICU, and stroke unit. CCNs in management positions were not included due to their potential impact on the reporting of their subordinates’ experiences [45].

According to the insights that emerged after the intermediate analysis of the first four focus groups, nine CCNs were purposively selected between January 2023 and April 2023 via a snowball sampling technique to deepen the understanding of the discussed topics from earlier focus groups [46]. For example, CCNs reported the detrimental influence of prehospital physician-staffed emergency care interventions on their health. Therefore, CCNs with similar and diverse experiences in prehospital physician-staffed emergency care interventions were recruited.

### Data collection

#### Data collection methods

Thorne’s interpretive descriptive approach was applied by conducting focus groups, which refer to a guided discussion with several people to explore ideas and perceptions about a specific topic from a multiplicity of views [47]. Conducting focus groups has several benefits, such as stimulating group dynamics, revealing deeper expressions of genuine feelings and beliefs, and enabling the acquisition of rich information in a cost-effective manner. Furthermore, the multiplicity of views during focus groups is useful to deepen the understanding of the complex social and psychological processes through which CCNs navigate their work-related demands, as these views could generate new ideas and perspectives that yield unexpected insights into the recalled experiences.

The research team consisting of experts in occupational health (EC, MK, and LBo), emergency nursing (LBi), and qualitative research (LBo) developed a semi-structured focus group guide (Table 1). This guide sought to explore the recalled experiences of CCNs in dealing with the physical and psychological influence of their work-related demands on their health and to identify strategies in which CCNs could mitigate this influence. The focus group guide used a deductive approach because of the preliminary exploration of the Job Demand-Control-Support model [34], the Effort-Reward Imbalance model [38], and the Sixth European Working Conditions Survey (EWCS) [48]. However, the focus groups were conducted with an open mind to identify new topics and to stimulate further questions that could contribute to the in-depth understanding of the CCNs’ recalled experiences [43]. As a result, the focus group guide became more focused when the transcripts were coded and preliminary ideas of the research team emerged [49].

**Table 1:** Focus group guide

Primary question	Possible probes
What aspects of your work influence your health?	• How do you experience: …exposure to physical activity at your workplace? …your working time quality? …a sense of responsibility? …patient-related stressful situations? …leisure-time physical activity in comparison to occupational physical activity? …exposure to emotional work-related demands? • Do you have the sense that your personality has changed due to your employment in a CCN ward?
How do you deal with certain health complaints at your workplace?	• Can you specify the nature and levels of health complaints at your workplace? • What influence has it had on you?
How do you perceive the efforts performed at your workplace?	• Does your employment at the CCN ward influence your private life? o How do you perceive this influence on your health?
Can you tell me about the perceived rewards for your delivered work?	
Can you tell me about the resources meaningful to you in your work environment?	• How do you experience social support from your co-workers and your supervisors (head nurse and physicians)? • Can you tell me more about the amount of control you have at your workplace? o What does more job control mean to you?
Can you tell me more about the amount of self-perceived commitment to the organisation?	• What are the consequences of this to you?
What would you like to change in your work environment to maintain a sustainable and health-promoting work environment to prevent health complaints?	

#### Data collection procedure

Between October 2022 and April 2023, six focus groups were held in a comfortable meeting room after lunchtime at the local hospital in Flanders (Belgium). Each focus group consisted of four to five CCNs from the same CCN ward and lasted uninterrupted for a maximum of 90 minutes, with an average duration of 68.75 minutes. The first 60 minutes were during working time, and the rest could be accounted as overtime. All focus groups were conducted by one master’s student in nursing science (LBi). The data collection process was supervised by an experienced qualitative researcher in occupational health (LBo) who provided feedback on the interview style. The master’s student was known superficially at the ED in the local hospital due to his previous nursing student work, which helped in understanding and contextualising the complexities and subtleties of the CCNs’ experiences. The interviewer wore clothes from the hospital to reduce the risk of interviewer bias. No observer was present during the focus groups. Because the participants were encouraged to share their experiences freely, the focus group guide was only implemented when the participants discussed topics irrelevant to this study, when a participant was too dominant, or when the discussion needed stimulation [45]. The interviewer sought to obtain input from all participating CCNs by asking open-ended and probing questions to introvert participants to elicit in-depth views. All focus groups were audiotaped with a smartphone and tablet.

### Data analysis

The audiotapes were transcribed verbatim and deleted afterwards. The data analysis process was based on the Qualitative Analysis Guide of Leuven, which guaranteed a cyclic process between data collection and data analysis to propose a conceptual framework [50]. The Qualitative Analysis Guide of Leuven consists of two crucial phases, namely, the preparation of the coding process by paper and pencil work and the actual coding process by using qualitative software [50].

First, two members of the research team (LBi and LBo) read the transcripts several times to obtain an in-depth understanding of the intricate details [18]. Second, both researchers wrote down memos and then developed a narrative focus group report for each focus group [50]. Third, concepts were drawn up to replace tangible or concrete experiences, which allowed the development of a conceptual scheme for each focus group. During this process, the same two researchers discussed and cross-checked the identified analytical and contextual concepts and sought to obtain a detailed understanding of the data [50]. This constant comparison process through inductive and interpretative reasoning allowed a within-case and across-case analysis to compare new concepts with earlier coded data so that similarities and differences in data could be identified and analysed [51–53]. Subsequently, the concepts were linked to relevant focus group fragments by using the QSR NVivo 12 software program. During this phase, data were further coded by combining concepts into groups of concepts based on emerging ideas and comparable meanings. These groups of concepts resulted in certain categories and were then divided into subcategories and main categories. The main categories were tested in the existing literature and rooted in the practical and theoretical knowledge of the research team after several intermediate meetings. Finally, the main categories were outlined in a conceptual framework, which represented the essential structure of the results. Data saturation was reached when no new dimensions or relationships emerged during the analysis, which was confirmed by conducting an additional focus group [52].

### Trustworthiness

The confirmability of the data was improved by applying different strategies. During the iterative process, the interview style and the questions arising from the focus group guide that could contribute to the in-depth understanding of the CCNs’ recalled experiences were peer-reviewed by the research team. Next, investigator triangulation was applied by two researchers with prior experience in the nursing profession (LBi and LBo) who analysed the transcripts independently and discussed the inductive code tree continuously. These transcripts and inductive code tree were then peer-reviewed by the entire research team at several intermediate meetings.

In addition, an audit trail with detailed information about the decisions made by the research team throughout the research process was documented to enhance the dependability and confirmability of the study [45]. This audit trail included descriptive interview notes, reflexive notes, methodological notes, and analytical notes. The development of reflexive notes was encouraged by sustaining transparent communication with the research team, which was stimulated because one research member was not familiar with occupational health, two research members were not a nurse, and one research member only had experience in the nursing profession in Switzerland [52]. Furthermore, the interviewer with experience in emergency nursing reflected on his personal values, opinions, and experiences, which cultivated awareness [43]. The audit trail also included a thick description of the setting, sample, and observations, supporting the transferability of the results. The Standards for Reporting Qualitative Research were implemented to enhance the quality of the reported data [54].

## Results

### Participants

The sample consisted of 37 CCNs, of which 27 CCNs participated in one of the six focus groups and ten CCNs could not participate due to organisational difficulties. Of those 27 CCNs, six were male and 21 were female, with a mean age of 36.07 years. Most CCNs worked in the ED (55.55%), with 77.78% of all included CCNs working full-time. Further sociodemographic characteristics of the CCNs are shown in Table 2.

**Table 2:** Sociodemographic characteristics of the CCNs (*N* = 27)

Sociodemographic characteristics	*N* (%)
**Age (years)**	
21–30	11 (40.74)
31–40	6 (22.22)
41–50	6 (22.22)
51–60	4 (14.82)
**Gender**	
Male	6 (22.22)
Female	21 (77.78)
**Highest educational degree**	
Bachelor	3 (11.11)
Bachelor and postgraduate in critical care nursing	23 (85.19)
Master	1 (3.70)
**Seniority as a CCN (years)**	
< 5	8 (29.63)
5–10	6 (22.22)
11–15	4 (14.82)
> 15	9 (33.33)
**Job time (%)**	
100	21 (77.78)
75–80	6 (22.22)
**Type of CCN ward**	
Emergency department	15 (55.55)
Intensive care unit	5 (18.52)
Stroke unit	5 (18.52)
Critical care mobile nursing team	2 (7.41)

N = number of participants, CCN = critical care nurse

### The interrelated categories

**Fig. 1:**
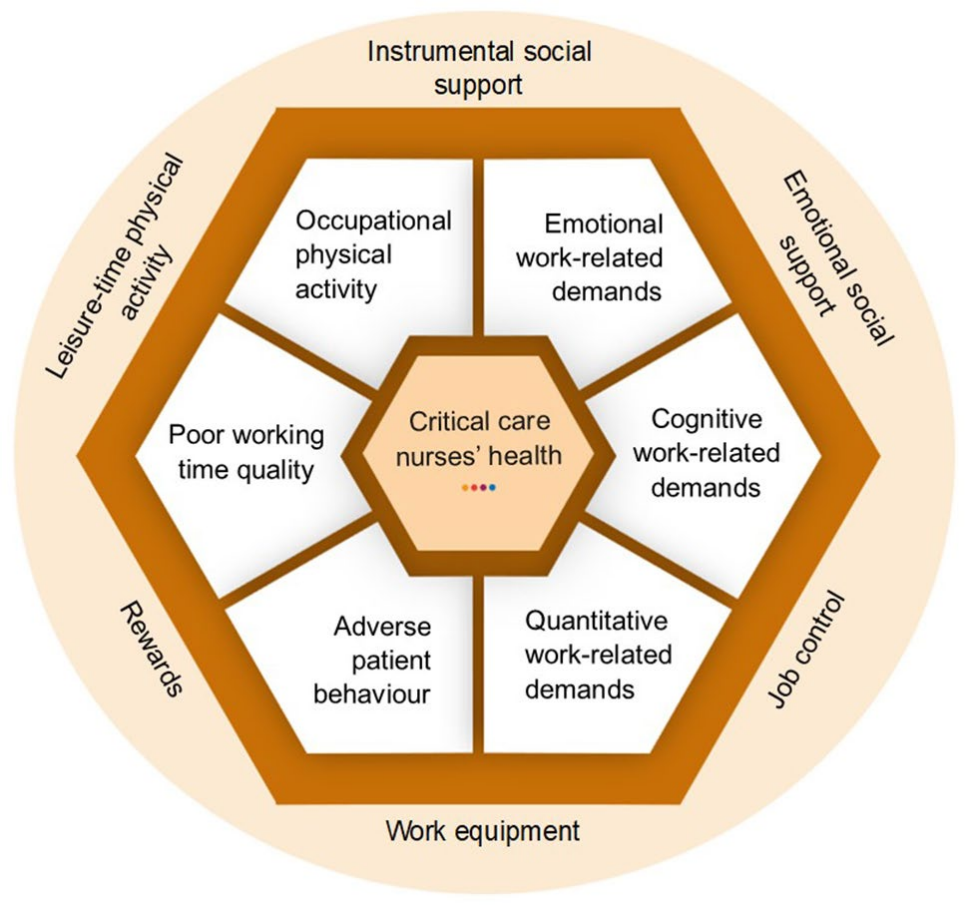
Conceptual framework inspired by the Job Demand-Control-Support model [34], Effort-Reward Imbalance model [38], and EWCS [48]

During iterative development, the influence of work-related demands on the participants’ health and mitigating strategies were identified. While being employed at a CCN ward, participants were continuously exposed to OPA, emotional, cognitive, and quantitative work-related demands, adverse patient behaviour, and poor working time quality. Exposure to such work-related demands was perceived as harmful and could lead to physical, mental, and psychosomatic complaints and increased turnover intention. Participants sought to mitigate the influence of work-related demands on their health by relying upon social support, job control, work equipment, rewards, and leisure-time physical activity. The results are outlined in the conceptual framework (Fig. 1). The central hexagon symbolises the consequences on CCNs’ health by surrounding work-related demands. The outer circle illustrates the applied strategies to mitigate adverse health outcomes.

The structuring of the results was inspired by the Job Demand-Control-Support model [34], the Effort-Reward Imbalance model [38], and the EWCS [48], and supported with exemplar citations referring to the specific participants along with the focus groups they belonged to (FG-P) [46].

#### Work-related demands

##### OPA

Participants experienced continuous exposure to OPA inside the hospital and during prehospital physician-staffed emergency care interventions. The included ED nurses were exposed to less OPA during the morning shift than ICU and stroke unit nurses. The most reported types of OPA were forward bending and isometric neck postures, prolonged standing, and long-distance walking. Forward bending and isometric neck postures were frequently required in various tasks performed, such as resuscitating, plastering, carrying heavy emergency coffers, tilling heavy patients in ambulance stretchers, and caring for intubated patients:

*For example applying a plaster, holding up a leg with one arm and your back being curved, I have already had instances where the day after I thought: ‘I had to hold up a leg of 50 kilos which made my arm hurt the day after’. (FG3-P3)*

##### Emotional work-related demands

Participants indicated the resuscitation of a child or family member, severe trauma victims, the announcement of cancer diagnosis to patients, and the high mortality rate as emotionally demanding:

*I have seen things during the COVID that I never want to see again. I found that terrible… Yes (…), that feeling of powerlessness. You had to go through it. How many people died alone? I held their hands, but I stood there alone in my alien outfit. Then you have to call the family and tell them that you didn’t leave them alone. Those family members started to cry and I cried with them. I have apologised for that… I found that a very heavy period, those first two months of COVID. In addition, those older persons who arrived and said: ‘You do not have to give the oxygen to us, give it to the younger persons’, and after two hours they were dead. (FG4-P4)*

In addition, the quality of management by supervisors was identified as a significant work-related demand among participants, as they reported feeling undervalued and unsupported, as well as experiencing a lack of empathy of their supervisors. Multiple CCNs claimed that the high number of telephone calls from their supervisors to provide shift coverage during off-job time contributed to this perceived poor management quality. FG1 and FG2 participants added that they felt the sense of being controlled by their supervisors via electronic patient records or checklists. The need for resilience, the changing work environment, and the lack of decision authority were further mentioned as significant demands in their role:

*They ask for your opinion when it has already been determined. That is something that often happens to us. They already decided on something and then asked us for the show like: ‘How do you think about it?’, but our opinion does not matter anymore. (FG3-P3)*

Furthermore, the adverse social behaviour from colleagues was cited as emotionally demanding by several participants. In particular, participating CCNs reported that interpersonal conflicts, such as working with nursing students, inexperienced colleagues or colleagues with whom the participants had a less good connection, contributed to an increased interdependency and the need to control the delivered care:

*You have colleagues you get completely stressed out by… Yes, because the way of working is completely different, that you cannot relate to them, that you cannot do anything right for them, whereas you have other colleagues where you feel each other. (FG4-P1)*

Finally, participants experienced a demand to perform without the ability to schedule a break and to be present at work during an illness because of their loyalty to colleagues:

*Recently, a colleague arrived with a kidney stone. She sat in the kitchen with an infusion of analgesics and started to work an hour and a half later. (FG1-P4)*

##### Cognitive work-related demands

Participants reported feeling highly vigilant throughout their shifts, especially when attending to unplanned care for critically ill patients. This required hypervigilance, combined with a lower presence of physicians, increasing their sense of responsibility. In addition, FG3 participants expressed being overwhelmed by the high amount of auditory stimulation they were exposed to:

*In the ICU, I do have more stress because of the responsibility in comparison with the ED. In the ED, the emergency physicians will do many things by themselves, whereas in the ICU, I am expected to do it by myself. In the ICU, you also have a lot more critical patients than in the ED, because in the ED, sometimes you have a lot of geriatrics, but there is nothing critical about it. Whereas in the ICU, if you have an unstable patient, you have to think and reason continuously. Then, again, that is tougher, the psychological aspect. (FG4-2)*

##### Quantitative work-related demands

Participants perceived the high work pace combined with telephone-related workflow interruptions, caused by managing the chaotic CCN ward and processing the high amount of medical orders, as harmful to their health. Furthermore, participating CCNs considered the need to carry out double work and inefficient work as significant demands in their role. As a consequence, multiple CCNs stated that more OPA was performed due to a lack of instrumental social support from colleagues:

*Sometimes you feel like you are behind the times. You have to do this and that and that and that. You have continuously, you are faced with something that is not feasible of care as you have been taught. In practice, that is not feasible. This is then shifted on a maximum of pressure (…). (FG4-P3)*

##### Adverse patient behaviour

Participants reported experiencing feelings of incongruence and dissatisfaction while providing care to self-referred non-urgent, dissatisfied, disrespectful, or aggressive patients:

*I sometimes feel unsafe, yes. Especially in the ED, very unsafe… Yes, I am roused and stressed. I put it away. I do not show it externally because I do not want the patient to realise this. Internally, this is something that eats you up. I feel I am tachycardic then. (FG4-P2)*

##### Poor working time quality

Participants highlighted the atypical working times as demanding due to working full-time in rotating shifts, on holidays, and during weekends:

*Those mixed evening shifts, morning shifts, night shifts, and day shifts… Yes, I stopped working full-time here because I could no longer cope with it. (FG4-P3)*

Furthermore, the highly commanded flexibility and poor working time arrangements were mentioned as significant work-related demands due to keeping up with all the refresher courses during off-job time, assisting in other nursing wards, dealing with unpredictable work schedules, and providing shift coverage when colleagues call in sick:

*I got a call an hour later from my nursing supervisor asking if I could work another night shift. However, I said: ‘It is my non-working weekend and again it is during my non-working weekend that I have to do a night shift’. Again, I was justifying myself and I thought: ‘Why am I doing that?’. They know my weaknesses and you gave in to one [supervisor], but the other one [supervisor] is also trying because maybe you will also give in to him. (FG5-P1)*

#### Consequences of work-related demands

##### Physical complaints

Participants reported experiencing musculoskeletal disorders, particularly after increased exposure to OPA during busy shifts. Multiple CCNs mentioned the most intense pain in the lower and upper back, neck, shoulders, knees, hips, or bilateral wrists. FG2 and FG3 participants also experienced inflammation in their feet, lateral epicondylitis, and restless legs at a young age:

*I have never, in the beginning, I did not suffer so much from that, but recently, I started having such restless legs from time to time <<< laughs>>>. In addition, then I think: ‘Oh so embarrassing because you are only 25 or 26 years old’. (FG3-P4)*

However, several participants suggested that they had developed musculoskeletal disorders more easily due to OPA compared to leisure-time physical activity. This distinction was attributed to the fact that OPA involves prolonged exposure to less intense physical activity and leisure-time physical activity involves shorter exposure to more intense physical activity:

*The physical work is more chronic (…), walking (…), or your arms or your back being strained… Whereas when you exercise that is very intense (…), your arms or your legs that you are training. (FG2-P3)*

Furthermore, FG4 participants experienced an increased risk of developing urinary disorders in terms of urinary tract infections and kidney stones. This increased risk was attributed to the lack of opportunities to drink while working and unhealthy toileting behaviours, such as delayed voiding while facing a high work pace. Moreover, participants stated that their rotating shift work and atypical working times led to irregular and unhealthy eating patterns, resulting in unintentional weight gain:

*I eat chips with a mandarin and a sandwich with chocolate, and minced meat. (FG3-P4)*

Last, participants reported that they had developed impaired sleep quantity in terms of insomnia, shortened or prolonged sleep duration, and increased sleep disturbances, which were probably caused by circadian rhythm disruption due to shift work:

*Yeah, especially if I had to switch from night to day rhythm. I was nauseous, intolerant, restless, rushed, unable to sleep, lying awake, not finding rest, being hungry when not being hungry. (FG4-P3)*.

##### Mental complaints

Participants mentioned experiencing challenges in detaching mentally from patient-related stressful situations, particularly when children or family members were involved. Further difficulties in detaching from work were attributed to the high number of consecutive working days, the changing work environment, the challenging weekend schedule/shift, and the considerable level of flexibility required of CCNs. Participating CCNs expressed that this lack of detachment contributed to their impaired sleep health, emotional exhaustion, concentration disorders, work-family interference, and alcohol consumption:

*I often need something like alcohol to just, truly, detach for a while <<< sighs>>>. My partner shares in the blows, but you are so overwhelmed at work and you come home with nine emails, a message from that one and a message from that one. On your day off again those emails, again those telephone calls, again… (FG5-P2)*

In addition, participants reported that they experienced work-related stress and more intense perceptions of OPA due to poor management quality, adverse social behaviour from colleagues, and working with nursing students. Multiple CCNs added that the refresher courses during off-job time, adverse patient behaviour, and the reported shortcomings in providing the best possible care to patients contributed to their perceived work-related stress, likely resulting in personal dissatisfaction, moral distress, carry over into their personal lives, and increased turnover intention:

*That satisfaction is completely overshadowed by the workload and the unsafe atmosphere at the ED. A stroke patient is located in the hallway and a person with epilepsy is located in the hallway, I am not satisfied when I come home. I just think: ‘No one died because of me in my care zone’. (FG5-P2)*

Furthermore, participants tended to experience feelings of agitation during exclusion from the multidisciplinary decision-making processes and due to the lack of social support from physicians and the confrontation with dissatisfied patients:

*We also do not understand why nurses were never involved in the development of patient rooms. I was part of the project group and when I measured everything and said it would not work for that, I got the reply: ‘Sorry, but it is too late, the rooms are already made and you cannot change that anymore’. (FG6-P4)*

In addition, participants perceived emotional exhaustion, which could lead to personality changes and reduced marital and life satisfaction:

*I do not know what all of you think about that, but everyone is sad at work. I feel that about myself too. (FG6-P2)*

Moreover, participants reported experiencing work-family interference and attributed this to the considerable level of flexibility required, the nature of shift work, and the presence of patient-related stressors. Because of this continuous interference, the included CCNs were not able to take care of their children, perform tasks at home, and spend time with family. This work-family interference caused work-related stress, emotional exhaustion, concentration disorders, impaired marital satisfaction, and a reduced perceived work ability among participants:

*So I also stopped working night shifts because of the work-life imbalance. From the moment I had my third child, I said: ‘This is no longer possible’. This caused tension in all possible areas, and then you have to make a choice and say that your private life comes first. It is almost not feasible to work full-time at the pace we work and in the circumstances we work. It is almost not feasible. (FG3-P1)*

Finally, participants expressed being subject to social isolation as a result of their demanded flexibility, shift work, and unpredictable work schedules:

*Yes, for example, I can no longer take dance classes because it is at a particular hour, and due to irregular shifts, I cannot guarantee that I can follow the class every week. So yes, too bad, but I cannot do my hobby anymore that I love to do. (FG3-P2)*

##### Psychosomatic complaints

Participants stated that the experienced emotional exhaustion and work-related stress led to unintentional weight loss, increased muscle tension, and migraine:

*I notice from myself that due to the emotional burden at work, I am starting to have physical complaints. For example, migraine, um yes, always being so tired, extremely losing weight, not being able to gain weight. (FG5-P1)*

Moreover, multiple participants expressed the physical effort of OPA and leisure-time physical activity as comparable, but the lack of decision authority and satisfaction that comes with OPA increased their risk of developing prolonged fatigue and emotional exhaustion, contributing to physical exhaustion:

*I can spend a whole day in my garden doing heavy work, then I come in [inside home] and I feel so energetic, fulfilled, and relaxed. However, when I come home from work, I feel so empty and drained of energy… The mindset here is already different. It [gardening] is also not an obligation. The work in the ED is an obligation… I can also feel that [physical activity during gardening] in my back and muscles, but still, I am not tired. (FG4-P3)*

Furthermore, repetitive exposure to work-related stress was seen by the participants as a main factor in developing heart palpitations and tachycardia:

*The moment I had tachycardia at triage due to enormous stress, no one cared from the physicians, except my two colleagues who then did take care of me. (FG5-P1)*

Additionally, participants experienced reduced sleep quality, which was attributed to work-related stress, emotional exhaustion, and lack of detachment. In particular, the participating CCNs faced excessive daytime sleepiness and nightmares:

*I went for a blood draw last week because my girlfriend said: ‘You should go for a blood draw because you are always tired, you always sleep around the clock and you would take another afternoon nap’. However, yes, everything was normal so the cause is probably my work. (FG5-P2)*

Last, participants reported that they had developed concentration disorders likely caused by work-related stress, prolonged fatigue, emotional exhaustion, and lack of detachment, increasing their risk of traffic accidents:

*I also nearly drove through a red light once. I had three to four prehospital physician-staffed emergency care interventions during one night and I was thinking of (…), anyways, I had to hit my brakes suddenly. (FG1-P1)*

##### Turnover intention

Participants stated that they tended to leave their CCN ward due to the high work pace, unsafe working conditions, work-family interference, and lack of social support from their supervisors:

*I have been in it [CCN profession] for more than 20 years now and I always said: ‘If it works out, I will stay in it until my retirement’… That you can stay employed until your retirement, I do not think that is possible anymore because of the current workload. (FG5-P5)*

#### Mitigating strategies

##### Social support

Participants reported instrumental social support from colleagues as a strategy to prevent the physical burden when dealing with OPA and to alleviate cognitive overload when coordinating a chaotic CCN ward:

*If I know it is a severely affected patient or someone who is somewhat corpulent and obese, I usually do go and ask the colleague: ‘Do you want to help me with turning this patient so I can wash his back?’. (FG2-P4)*

Moreover, the included CCNs indicated that emotional social support from supervisors and colleagues reduced their work-related stress by putting work-related demands into another perspective. As a result, participants carried less emotional and cognitive work-related demands over into their personal life, improving their mental well-being and marital satisfaction. Multiple CCNs added that ventilating to a self-employed psychologist or a family member who also works in healthcare helped them prevent emotional exhaustion and burnout:

*Listening, giving advice, helping you, cheering you up, coming to help you unasked (…). Just asking if they can do something, for instance. Often they cannot do anything, but just the question they ask does wonders. (FG4-P1)*

##### Job control

Participants emphasised a high amount of skill discretion due to accommodative access to refresher courses, which contributed to their sense of safety and resulted in less work-related stress and more job satisfaction. Concerning decision authority, multiple CCNs considered the perceived amount of control to schedule their holidays and take up overtime as an important motivator to cope with work-related demands. Additionally, participants stated that the authority to schedule a break at work was needed to recover mentally and physically during periods of high work pace:

*It feels good if you can recuperate for once. If you now say like, for example, in certain night shifts, you have finished your patient care, and at midnight or 1 AM, you say: ‘Come, let us drink a coffee’. That you can <<< blows out>>>. This is just for 15 minutes because you still have to do… (FG1-P5)*

##### Work equipment

Participants expressed that work equipment to transfer patients, such as the HoverMatt®, sliding sails, and patient lifts, alleviated the physical burden of OPA. Nevertheless, several CCNs reported shortcomings in ergonomic work equipment to address OPA during prehospital physician-staffed emergency care interventions. In addition to these shortcomings, work equipment to transfer patients was not used to its full potential while facing a high work pace. Furthermore, participants disclosed that adjustable hospital stretchers, ergonomic shoes, and chairs with adaptability for taking blood samples were beneficial in preventing physical complaints. Participants in FG1 and FG3 added the benefits of compression stockings, analgesics, and magnesium to avoid restless legs:

*And especially if you work night shifts, the restless legs that you have when you get into your bed. Now, I no longer have that <<< looks at compression stockings>>>. (FG1-P4)*

##### Rewards

Participants perceived the patients’ gratitude, wages, job security, equal social benefits, career prospects, and off-job time as helpful to cope with the required efforts at work:

*That you have been able to do your job the way you want and if you build up a good relationship with your patient who you feel you have been able to help him both physically and mentally through the difficult period, then this does give you satisfaction, uhm. (FG2-P3)*

##### Leisure-time physical activity

Participants indicated leisure-time physical activity as a strategy to detach mentally from work:

*I exercise every day and that just helps me more, I am more relaxed compared to when I do not exercise. (FG1-P2)*

## Discussion

Participants were exposed to OPA, emotional, cognitive, and quantitative work-related demands, adverse patient behaviour, and poor working time quality. In response to these work-related demands, participants employed various strategies for mitigation, including seeking social support, exerting control over their work, utilising appropriate equipment, recognising rewards, and engaging in leisure-time physical activity. Throughout the following discussion, the results were compared with traditional quantitative frameworks used in research on psychologically healthy work to investigate if these frameworks still comprise all essential factors influencing CCNs’ work-related health.

A key finding of this study was the continuous exposure to a high amount of OPA. However, contrary to Aleid et al. [55], this study sample identified differences in exposure to OPA between the different participating CCN occupations. This result could be attributed to two organisational factors of the local hospital. First, the hospital’s patient occupancy rate is normally lower during the morning at the ED compared to the ICU and stroke unit. Second, the participating ICU and stroke unit nurses had their work equipment to deal with OPA more closely available in the patient room, while ED nurses did not [9]. In contrast to Clays et al. [56], however, this study also emphasised the psychosocial work environment as an influencer of exposure to OPA. This result could be explained by the participating CCNs experiencing adverse social behaviour from colleagues with whom they had a less good connection, resulting in them receiving less instrumental social support and having to perform more OPA alone. Another possible explanation could be that these CCNs were subject to more OPA due to the lack of authority to question medical orders given by physicians. This may be attributed to the experienced patriarchal physician-nurse relationship and the financial incentive of diagnostic tests for physicians due to the fee-for-service payment system in Belgium. Because of the exposure to OPA, the CCNs in this study reported experiencing musculoskeletal disorders, which corroborates the results of previous studies among CCNs [1, 57]. Despite several risk management strategies across the nursing profession to reduce the risk of developing musculoskeletal disorders, exposure to side-bending postures during prehospital physician-staffed emergency care interventions is not decreasing [58, 59]. From a theoretical perspective, OPA is widely covered by the physical job demands subscale of the Job Demand-Control-Support model [34], the effort subscale of the Effort-Reward Imbalance model [38], and the physical environment index of the EWCS [48].

Exposure to emotional work-related demands related to exclusion from multidisciplinary decision-making processes and providing inappropriate care to patients and their relatives resulted among the participants in moral distress and emotional exhaustion. Consistent with Azoulay et al. [40], this mental burden can be considered an important factor in developing burnout. As a consequence, participants tended to experience unintentional weight loss, migraines, personality changes, job dissatisfaction, and increased turnover intention. Concerning personality changes, previous research has noted that 38.6% of South Korean ICU nurses were characterised by a Type D personality in terms of anxiety, depression, and inappropriate worrying [60]. However, CCNs in this study also experienced less empathy towards their patients, and remarkably less empathy towards their partners and friends. Despite the major influence of emotional work-related demands on CCN’s health, these demands are solely covered by the EWCS [48].

Our results indicate that exposure to cognitive work-related demands during employment at a CCN ward is essential to consider when evaluating CCNs’ health. Previous research has indicated that the continuous solving of unforeseen problems can contribute to self-development at work [48]. However, consistent with Bolliger et al. [46], the included CCNs perceived this continuous problem-solving as stress-inducing. An increasing amount of evidence suggests that the required cognitive hypervigilance of CCNs can increase the risk of concentration disorders and may lead to medical errors [10, 11]. This increased risk of medical errors was not demonstrated by this study, which could be due to socially desirable answers during the focus groups. Cognitive work-related demands are part of the effort subscale of the Effort-Reward Imbalance model [38] and the skills and discretion index of the EWCS [48].

Participants underscored that exposure to quantitative work-related demands in terms of high work pace, workflow interruptions, and inefficient work reduced their attention and sleep health due to work-related stress, which is well supported by evidence [10]. Multiple participating CCNs experienced reduced subjective sleep quality, disrupted sleep duration, and increased sleep disturbances, which were associated with an increased risk of traffic accidents, and are in line with Smyth’s [61] Pittsburgh Sleep Quality Index. According to the theoretical models, quantitative work-related demands are covered by the demands subscale of the Job Demand-Control-Support model [34], the effort subscale of the Effort-Reward Imbalance model [38], and the work intensity index of the EWCS [48].

Consideration is required concerning the influence of working time quality on CCNs’ health. Regarding the working time quality index of the EWCS [48], the combination of atypical working times and family role demands was perceived by participants as detrimental to their health and marital life. A possible explanation for this might be that most participating CCNs were aged between 25 and 35 years, which is seen as the most interesting period for career development, marriage, and raising children [31, 62, 63]. Furthermore, in line with the EWCS [48], participants who were informed at short notice of adaptations in their work schedule tended to experience a lack of detachment, work-family interference, and social isolation. However, previous research has shown that male workers are more likely to develop low back pain due to work-related demands when they experience work-family interference [64]. Given these results, nursing supervisors should give more consideration to the risk factors for work-family interference in risk management strategies to prevent the development of musculoskeletal disorders. The dimensions of the working time quality index are not considered by the Job Demand-Control-Support model [34] or by the Effort-Reward Imbalance model [38].

This study identified workplace social support as a psychosocial moderator of the development of emotional exhaustion due to stress-inducing work-related demands. In line with Sampei et al. [65], participants reported that they had developed emotional exhaustion when they faced high exposure to work-related demands with low levels of social support. In contrast to Clays et al. [35], however, no evidence of the buffering potential of social support on the development of coronary heart diseases due to OPA was detected. From a theoretical perspective, workplace social support is widely mentioned in the Job Demand-Control-Support model [34], the Effort-Reward Imbalance model [38], and the EWCS [48].

Concerning skill discretion, access to training opportunities among the European workforce improved by 12% in 2015 compared to 2005 [48]. This finding is consistent with this study, in which accommodative access to refresher courses contributed to the participants’ sense of safety at work. However, the amount of flexibility required to be present in the refresher courses during off-job time was likely to induce work-related stress and work-family interference. Regarding decision authority, participants experienced exclusion from multidisciplinary decision-making processes and had fewer opportunities to schedule a break at work. This result is consistent with the EWCS [48] stating that only a scarce 33% of European subordinates were involved by their supervisors in decision-making processes influencing their work [46]. Surprisingly, the Job Demand-Control-Support model [34] was found to measure job control solely on positively perceived decision authority [46].

The included CCNs expressed the mitigating influence of wages, career prospects, and job security in regard to coping with work-related demands. According to the earnings index of the EWCS [48], 39% of the European workforce agreed that their employment offers prospects that are beneficial for career advancement. This is in line with this study, in which participants perceived that being employed at a CCN ward contributed to their professional development. From a theoretical perspective, the Effort-Reward Imbalance model [38] includes the rewards subscale in terms of money, esteem, and security/career opportunities.

In light of the discussed theoretical models, some show additional shortcomings. Although the participants’ health was influenced by patient-related stressful situations, poor management quality, and the experienced demand to perform, these emotional work-related demands are not considered by the Job Demand-Control-Support model [34] or by the Effort-Reward Imbalance model [38]. In addition, the Job Demand-Control-Support model [34] does not pay attention to the work-family interference concept caused by the considerable level of required flexibility, the nature of shift work, and the presence of patient-related stressors. However, the Effort-Reward Imbalance model partially conceptualises work-family interference as overcommitment [46, 48]. Finally, the Job Demand-Control-Support model [34] does not include the rewards subscale in terms of patients’ gratitude, wages, job security, equal social perks, career prospects, or off-job time. Thus, solely the EWCS [48] covers a wide range of work-related demands that are perceived as harmful according to this study sample.

### Limitations

The inclusion of only one hospital may have provoked selection bias and might hinder the transferability of the results to other CCNs employed in similar work environments. Furthermore, the scheduled focus groups with the ICU nurses were frequently cancelled at short notice due to seasonal epidemics and changing work schedules. In addition, the stroke unit’s nursing team is characterised by a limited number of nurses, and therefore, it was only possible to organise one focus group. As a consequence, the subgroup of ICU and stroke unit nurses was small, and data saturation concerning sampling remains debatable. Another limitation is the possible occurrence of healthy worker effect bias [66], as nurses on sick leave may have felt impeded from participating. During each focus group, essential observations could have been missed due to the absence of an observer. Additionally, interviewer bias may have occurred due to the moderator’s pre-existing superficial relationship with the ED nurses. However, the research team is convinced that the CCNs employed in the local hospital were not hampered from engaging and that this relationship stimulated them to share their deep-rooted feelings and perceptions.

### Implications for practice

The results of this study include several recommendations for practice, structured by the developed conceptual framework. In particular, the identified and assessed physical and psychosocial risk factors can be integrated into the current risk management strategies. This is crucial as existing risk management strategies often overlook the consideration of multiple risk factors. Concerning OPA, more ergonomic emergency coffers could be provided to prevent side-bending postures during prehospital physician-staffed emergency care interventions. To tackle emotional work-related demands, nursing supervisors should provide vertical trust, job security, transparent communication, decision authority, and social support to their employees, thereby mitigating the perceived influence of work-related demands [46]. Addressing job security, the meta-analysis of Kim and von dem Knesebeck [67] demonstrated that employees without job security had 29% more risk of developing depressive symptoms compared to employees with job security. Moreover, Mazzetti et al. [68] underscored the need for organisations to provide a leadership programme in which supervisors learn appropriate coaching strategies, enhancing proximal factors such as job satisfaction and commitment. In reference to cognitive and quantitative work-related demands, greater efforts are needed to ensure a sufficient and uninterrupted recovery time between shifts, to provide breaks without interruptions and to reduce the demand to perform [69]. With respect to adverse patient behaviour, the risk of mental complaints and work-related stress can be reduced by assigning a psychologist who educates CCNs on how to cope with patient-related stressful situations. Furthermore, nursing supervisors can improve poor working time quality by implementing forward and rapidly rotating work schedules to impede the development of circadian rhythm disruption [25]. In addition, schedule flexibility should be guaranteed by introducing the principles of self-scheduling to provide more control over working time, prevent work-family interference, and reduce the risk of circadian rhythm disruption [25].

### Implications for research

Considerably more work will need to be done to determine the long-term moderating effects of psychosocial job resources by implementing longitudinal research designs. Additionally, further studies need to be carried out to establish the modernisation of traditional quantitative frameworks used in research on psychologically healthy work, in which they explore the role of psychosocial and organisational factors in more detail. Concerning the modernisation of these frameworks, the influence of individual work-related demands on CCNs’ health is well-known according to recent evidence. However, research on the influence of multiple intertwined work-related demands on the health of CCNs remains scarce. As increasing research employs latent profile analyses, the interdependence of job factors becomes evident. Therefore, future research should investigate how multiple work-related demands interact or manifest in certain combinations on CCNs’ health.

## Conclusions

This qualitative study identified that the participants’ health was challenged by work-related demands that are not entirely covered by the traditional quantitative frameworks used in research on psychologically healthy work. In particular, CCNs included in this study were exposed to OPA, emotional, cognitive, and quantitative work-related demands, adverse patient behaviour, and poor working time quality. In response to these demands, these CCNs employed various strategies for mitigation, including seeking social support, exerting control over their work, utilising appropriate equipment, recognising rewards, and engaging in leisure-time physical activity. Therefore, future studies should explore the role of psychosocial and organisational factors in more detail. In conclusion, this study recommends the development of an employee-centric work environment by providing sufficient risk management strategies, schedule flexibility, uninterrupted off-job recovery time, and positive management to guarantee extended healthy working lives among the CCN workforce.

## Data Availability

The datasets used and/or analysed during the current study are available from the corresponding author upon reasonable request.

## Abbreviations

CCNs: Critical Care Nurses
EWCS: European Working Conditions Surveys
ED: Emergency Department
ICU: Intensive Care Unit
OPA: Occupational Physical Activity
QUAGOL: Qualitative Analysis Guide of Leuven
